# Expanding horizons: lung transplantation for non-IPF interstitial lung diseases

**DOI:** 10.1186/s12890-024-03291-4

**Published:** 2024-10-02

**Authors:** Sevinc Citak, Ertan Saribas, Ayse Nigar Halis, Fatma Feyza Alkilic, Murat Ersin Cardak, Mustafa Vayvada, Ahmet Erdal Tasci

**Affiliations:** 1 Department of Thoracic Surgery, Kartal Kosuyolu High Specialization Education & Research Hospital, Istanbul, Turkey; 2 Department of Pulmonology, Kartal Kosuyolu High Specialization Education & Research Hospital, Istanbul, Turkey

**Keywords:** Non-IPF ILD, Lung transplantation, Silicosis, Langerhans cell histiocytosis X, Pleura parenchymal fibroelastosis

## Abstract

**Objective:**

Interstitial lung diseases (ILDs) are diverse pulmonary disorders marked by diffuse lung inflammation and fibrosis. The variability in characteristics and treatment approaches complicates diagnosis and management. In advanced cases requiring transplantation, determining indications and selecting suitable candidates presents additional challenges.

**Methods:**

Of all patients with non-IPF ILD between December 2016 to December 2022 were analyzed retrospectively. Patients were categorized into two groups: transplanted patients and deceased patients on the waiting list. Clinical data and survival outcomes were compared between groups.

**Results:**

Of the 43 patients, 20 underwent lung transplantation while 23 died awaiting transplantation. Waiting list mortality was 53.4%, with median waiting times similar between groups (3 months for transplant patients and 6 months for those on the waiting list). There were no significant differences between groups in age, gender, height, BMI, 6-minute walk test (6MWT), or forced vital capacity (FVC). The prevalence of pulmonary hypertension (PH) was 76.7% in right heart catheterizations, similar in both groups. One single and 19 bilateral lung transplants were performed. Overall, 13 of the 20 patients survived to discharge from the hospital. One-year mortality was 7/20 (35%). The median follow-up was 34 months, with a 1-year conditional survival of 90.9% at 3 years and 70.7% at 5 years.

**Conclusions:**

This study underscores the importance of further research into non-IPF ILDs. Lung transplantation remains a viable option that can significantly enhance both the quality and longevity of life for patients with advanced ILD.

## Background

Interstitial lung diseases (ILDs) encompass a heterogeneous collection of pulmonary disorders marked by diffuse inflammation and fibrosis within the lung interstitium. These conditions, which can present acutely or progress chronically, result in varying degrees of lung parenchymal damage, structural deterioration, and impaired pulmonary function [1]. The clinical manifestations and progression of ILDs are highly variable, contingent on the specific subtype and underlying etiology. Generally, these disorders culminate in the scarring or fibrosis of lung tissue, posing significant challenges for treatment and management.

The American Thoracic Society and the European Respiratory Society categorize ILDs into four principal groups: (1) ILDs from known causes or exposures (e.g., drugs, occupational exposures, connective tissue diseases); (2) idiopathic interstitial pneumonias; (3) granulomatous ILDs (e.g., sarcoidosis, certain infections); and (4) rare forms of ILDs (e.g., histiocytosis, neurofibromatosis) [2]. This classification framework is pivotal for guiding treatment strategies and managing the diverse etiologies, pathophysiological mechanisms, and clinical trajectories of ILDs.

Each ILD category exhibits distinct characteristics and requires tailored therapeutic approaches, complicating both diagnostic and treatment processes. In advanced ILD cases necessitating lung transplantation, establishing clear indications for transplantation and selecting appropriate candidates becomes particularly challenging. Transplantation criteria typically hinge on the patient’s overall health, the severity of respiratory failure, the rate of disease progression, and the inadequacy of alternative treatments. Given the heterogeneity of ILDs, transplantation indications must be personalized, although they generally converge on common clinical thresholds [3].

Significant research on idiopathic pulmonary fibrosis (IPF) has expanded our understanding of its definition and treatment, establishing lung transplantation as a critical advanced therapy. However, studies focusing on lung transplantation for other ILD subtypes remain sparse. This paucity of research is partly due to the considerable variability among ILDs, with some subtypes being less common or less well-characterized than IPF. Consequently, there is limited understanding of the transplantation needs and outcomes for non-IPF ILDs.

Despite these challenges, the proportion of lung transplants performed for ILD globally rose to 40.5% in 2017, with 32.4% specifically for IPF.^3^ Survival rates also vary significantly among ILDs: for example, the 1-, 3-, and 5-year survival rates for hypersensitivity pneumonitis were 96%, 89%, and 89%, respectively, whereas for Langerhans cell histiocytosis X, the corresponding rates were 76%, 63%, and 57%. Such variability underscores the necessity for further data collection to elucidate the transplantation outcomes for this diverse patient population [4, 5].

To enhance our understanding of the non-IPF ILD cohort, we conducted a descriptive analysis at our center, followed by a retrospective analysis to identify pre-transplant characteristics impacting waiting list mortality and post-transplant outcomes. This study aims to contribute to the limited but growing body of knowledge on lung transplantation for ILDs beyond IPF, ultimately improving patient management and outcomes in this complex and varied subgroup.

## Methods

A single-center retrospective analysis was conducted on patients who presented to our lung transplant clinic between December 2016 and December 2022 and were listed for lung transplantation due to interstitial lung disease. For the diagnosis of ILDs, patient history, radiological images, disease-specific blood tests, and, when available, transbronchial or lung biopsies were reviewed to establish or confirm the diagnosis. Eighty-one patients were listed with an ILD diagnosis. Of these, 28 patients were diagnosed with IPF and were subsequently excluded from the study. A total of 53 patients were diagnosed with non-IPF ILD. Patients with non-IPF ILD were included in the study after at least 18 months of follow-up, during which their progress was observed. Four patients were listed but later removed for various reasons. Additionally, six patients still on the waiting list were not included in the two groups due to the uncertainty of their clinical conditions. Consequently, these patients were excluded from the study to ensure adherence to the study protocols.

The remaining patients were divided into two groups: “transplanted patients” (TP) and “deceased patients on the waiting list” (DPWL). Patients were analyzed for their diagnoses, demographic data including height (cm), weight (kg), body mass index (BMI; kg/m^2), and time spent on the waiting list (months), as well as laboratory tests measuring renal function (serum creatinine [mg/dL]) and liver function (total bilirubin [mg/dL]). Additionally, data on lung function tests such as the 6-minute walk test (6MWT; distance [m]) and forced vital capacity (FVC) were recorded at the time of listing. Echocardiography and cardiac catheterization were examined to assess cardiological functions. The “transplant patients” and “deceased patients on the waiting list” groups were compared based on these data and evaluated for survival.

### Ethics approval and consents to participate

The study protocol was approved by the Ethical Committee of Kartal Kosuyolu High Specialization Education & Research Hospital (No: 2024/13/870). This study was conducted according to the Helsinki principles, patients signed informed consent for participation, and nothing invasive of patients’ privacy was done.

The study was a human organ/tissue transplant study, as it involved lung transplantation for patients with interstitial lung diseases other than IPF. We confirm that organs/tissues were not taken from prisoners. Each organ/tissue was taken from volunteer donors. And when these donors became brain dead, Written informed consent was also obtained from their first-degree relatives.

And also, written informed consent was obtained for publication of this original article and all accompanying images from all patients who underwent transplantation and from whom that tissue/organ was taken.

### Statistical analysis

The distribution of quantitative data was analyzed using the Shapiro-Wilk test. Data were expressed as a median (minimum–maximum). Mann–Whitney U and Student T tests were used to compare characteristics between groups TP and DPWL. Qualitative data were compared using the Chi-Square and Fisher’s exact tests. The Kaplan–Meier method was used for survival analysis. A value of *P* < 0.05 was considered to be statistically significant. The analysis was performed using the SPSS for Windows software (IBM, Armonk, NY, USA).

## Results

**Fig. 1:**
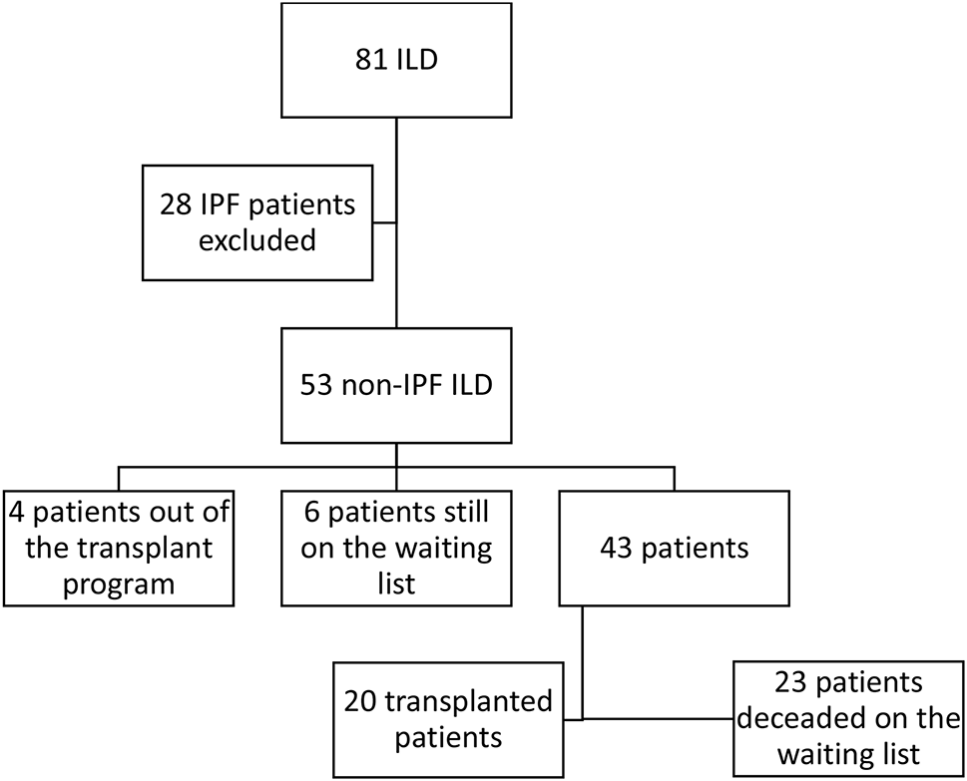
Flow diagram of study participants

Between December 2016 and December 2022, 81 patients with ILD were listed for lung transplantaion Between December 2016 and December 2022, 81 patients with ILD were listed for lung transplantation at Koşuyolu High Specialization Education and Research Hospital. Twenty-eight patients diagnosed with IPF were excluded from the study. Additionally, four of the 53 patients diagnosed with non-IPF ILD were excluded due to deterioration during the follow-up while on the lung transplant waiting list. Furthermore, six patients who were still on the lung transplant waiting list were excluded because their condition had not been finalized. Of the 43 patients reviewed, 20 received lung transplants, and 23 died while on the waiting list (Fig. 1).

The most common diagnoses among patients were fibrotic nonspecific interstitial pneumonitis (NSIP) (14 [32.5%]), silicosis (4 [9.3%]), and sarcoidosis (4 [9.3%]). The study comprised 34 males (79.1%) and 9 females (20.9%). The median age was 50 years (14–63 years). The median BMI was 23 kg/m^2^ (13–29 kg/m^2^), and the median height was 169 cm (140–187 cm). Functional testing revealed a median 6MWT of 260 m (35–475 m), with 9 patients (21%) demonstrating a maximal 6MWT of less than 180 m. The median forced vital capacity (FVC) was 43% predicted (17-87%) based on the listing pulmonary function test. Echocardiography revealed right ventricle dilation in 17 patients. The median tricuspid annular plane systolic excursion (TAPSE) was 2 mm (1.1–3 mm). Age distribution between groups TP and DPWL was 41 vs. 54 (*p* = 0.086) (Table 1).

**Table 1:** Demographic data of patients

	Total *N* = 43 (%)	Transplanted Patients *N* = 20 (%)	Deceased patients on waiting list *N* = 23 (%)	*p*
Age *median* (years)	50.0 (14–63)	41.5 (14–63)	54.0 (22–62)	0.086
Gender				
Female Male	9 (20.9) 34 (79.1)	3 (15) 17 (85)	6 (26) 17 (73)	0.467
Blood type (%)				
A B AB O	26 (60) 4 (10) 2 (5) 11(25)	12 (60) 2 (10) 1 (5) 5 (25)	14 (61) 2 (9) 1 (4) 6 (26)	
Height (*cm*)	169 (140–187)	171.5 (159–187)	167 (148–176)	0.704
BMI (kg/m^2^)	23.3 (13–29)	21.6 (13/29)	25 (15–29)	0.067
Treatment at the listing time				
Antifibrotic agent Prednisolone	7 21	3 7	4 14	0.704 0.258
6 MWT (m) < 180 m (%)	260 (35–475) 9 (21)	270 (35–475) 4 (20)	260 (60–440) 5 (21)	0.686 0.690
FVC %	43.0 (17–87)	43.5 (18–87)	43.0 (17–57)	0.285
Primary lung disease				
Fibrotic NSIP Sarcoidosis Silicosis CTD-ILD HP PPFE GVHD Unclassifiable Alveolar proteinosis Langerhans cell histiocytosis X Postcovit pulmonary fibrosis Pulmonary ossification	14 (32.5) 4 (9.3) 4 (9.3) 3 (6.9) 3 (6.9) 3 (6.9) 3 (6.9) 3 (6.9) 2 (4.6) 2 (4.6) 1 (2.3) 1 (2.3)	4 (20) 2 (10) 3 (15) 1 (5) - 2 (10) 2 (10) 2 (10) 1 (5) 1 (5) 1 (5) 1 (5)	10 (43.4) 2 (8.7) 1 (4.3) 2 (8.7) 3 (13) 1 (4.3) 1 (4.3) 1 (4.3) 1 (4.3) 1 (4.3) - -	
Echocardiography				
TAPSE (*mm*) RV dilation	2 (1.1-3) 17 (39)	1.9 (1.5-3) 9 (45)	2 (1.1–2.8) 8 (34)	0.502 0.494
Rigth heart catheterization				
PABs PABm PVR (WU) CI CO PH (%)	43 (19–90) 22 (10–60) 3.1 (0.6–22.4) 2.4 (1.32–6.9) 4.3 (2.05–9.4) 33 (76)	48 (22–85) 23.5 (12–51) 3.7 (1.1-9) 2.2 (2-6.9) 4.2 (3.2–9.4) 15 (75)	37 (19–90) 22 (10–60) 2.7 (0.6–22.4) 2.4 (1.3–4.2) 4.4 (2.05–6.6) 18 (78)	0.267 0.407 0.300 0.463 1 1.000
Waiting time on the list *mounth*	5 (1–52)	3 (1—21)	6 (1–52)	0.150
Serum creatinine, mg/dL	0.65 (0.5–1.17)	0.72 (0.29–1.17)	0.62 (0.44–1.13)	0.257
Serum bilirubin mg/dL	0.5 (0.21–1.6)	0.5 (0.21–1.5)	0.50 (0.3–1.6)	0.434

BMI, body mass index; 6 MWT(m), six minutes walking test; FVC, forced vital capacity; NSIP, nonspecific interstitial pneumonitis; CTD-ILD, connective tissue disease related interstitial lung disease; HP, hypersensitivity pneumonitis; PPFE, pyloroparenchymal fibroelastosis; GVHD, graft versus host disease; TAPSE, tricuspid annular plane systolic excursion; RV, right ventricle; PABs, systolic pulmonary arterial pressure; PABm, mean pulmonary arterial pressure; PVR, pulmonary vascular resistance; CI, cardiac index; CO cardiac output; PT pulmonary hypertension

There was no significant difference between groups in terms of patient age, gender, height, BMI, 6MWT, and FVC. The prevalence of pulmonary hypertension (PH) in right heart catheterization was 76.7% (33/43). The distribution was similar in both groups. The waiting list mortality was 53.4% (23/43). The median waiting time on the list was similar for groups TP and DPWL with 3 months (1–21 months) and 6 months (1–52 months), respectively.

Twenty lung transplants were performed, consisting of one single and 19 bilateral procedures. The clamshell incision was utilized in all patients. However, in the case of the patient with scleroderma, a single lung transplantation was performed due to significant adhesions and unstable hemodynamics. The median durations of mechanical ventilation and intensive care unit stay were 2 days (1–14 days) and 5 days (4–63 days), respectively. The average ischemia time was 342 min (210–480 min) for the first lung transplantation and 502 min (360–576 min) for the second lung transplantation.

Intraoperative Extracorporeal Membrane Oxygenation (ECMO) was utilized in 11 cases (55%). Eight of 11 patients required ECMO because of pulmonary hypertension (PH). Three patients without PH received preoperative ECMO, one of whom had a lobar transplant, and the other two received ECMO due to perioperative hemodynamic instability. Eight out of 11 patients exhibited good respiratory parameters and hemodynamic stability after lung transplantation; thus, ECMO support was terminated. Three patients failed ECMO weaning due to hemodynamic reasons. Two patients had bleeding complications, and one suffered from cardiac failure, resulting in unstable hemodynamics.

**Fig. 2:**
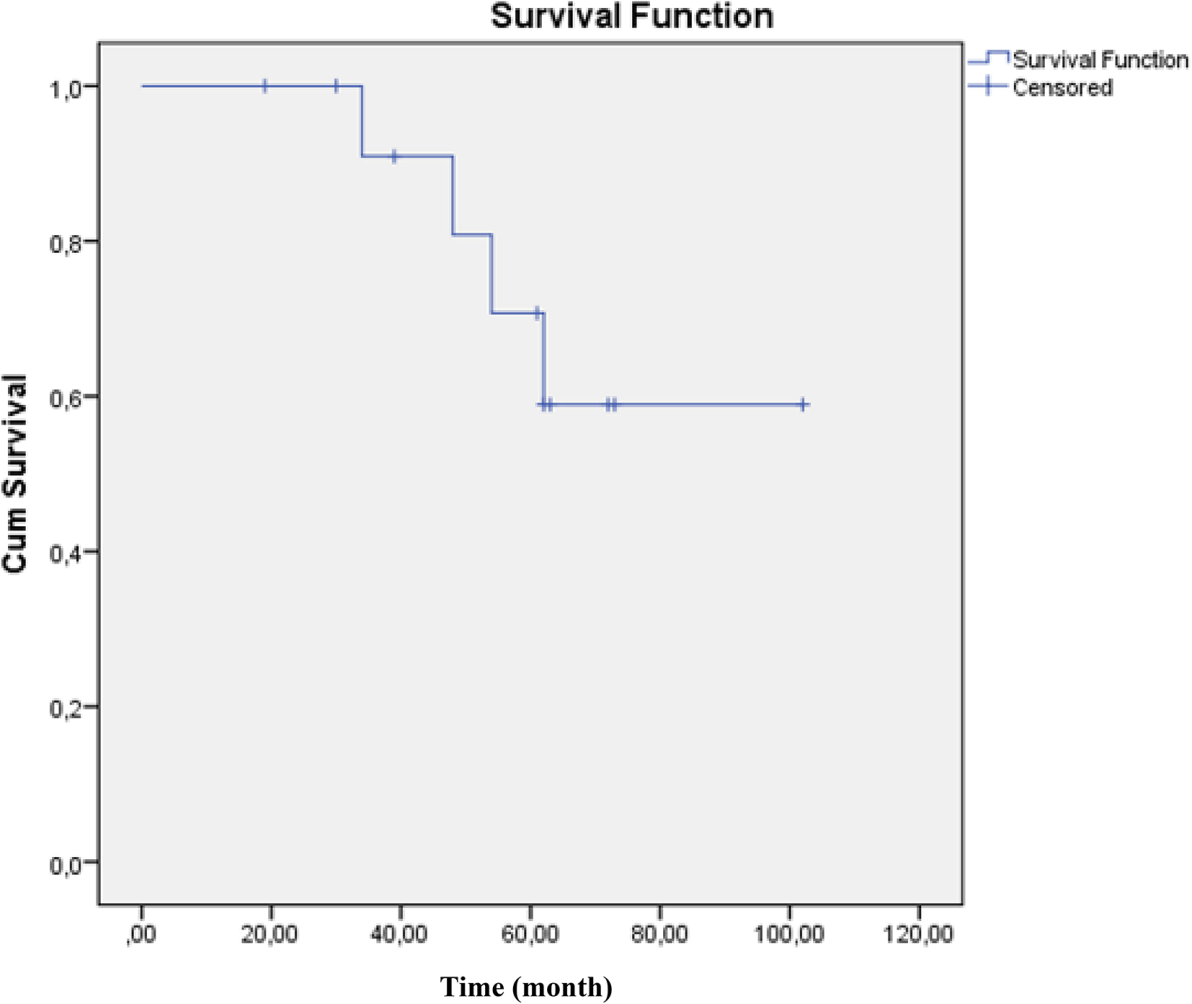
Cumulative survival

Overall, 13 of the 20 patients survived to discharge from the hospital. One-year mortality was 7/20 (35%). The median follow-up period was 34 months (1–102 months). Causes of death included bleeding in two patients, sepsis in two patients, cardiac failure in two patients, and cerebrovascular disease in one patient (Table 2). The 1-year conditional survival (i.e., the probability of surviving the next year) was 90.9% at 3 years and 70.7% at 5 years (Fig. 2).

**Table 2:** Intraoperative data and outcomes (*n* = 20)

Transplantation type	
Bilateral Single	19 1
Ischemic time minutes	
First lung Second lung	342 (210–480) 502 (360–576)
Intraoperative ECMO	11 (55%)
Postoperative ECMO	6 (30%)
Severe PGD	6 (30%)
Mechanical ventilation, days	2 (1–14)
Tracheostomy	7 (35%)
ICU stay, days	5 (1–40)
Hospital stay, days	14 (4–63)
Hospital mortality	7 (35%)
Cause of mortality	
Bleeding Sepsis Cardiac failure Cerebrovascular disease	2 2 2 1
Three years survival (cumulative)	90.9%
Five years survival (cumulative)	70.7%

## Discussion

Our study revealed a waiting list mortality rate of 53.4%, significantly exceeding the rates reported in the literature [6–8]. In contrast, data from a 2022 report in the USA indicated that waiting list mortality for lung transplants was approximately 13% [9]. This disparity can be primarily attributed to the limited availability of suitable donors in our country compared to more experienced nations. Additionally, the shorter life expectancy of our patients while on the waiting list further complicates organ transplantation efforts. This dual challenge of donor scarcity and reduced patient survival underscores the complexities of managing and treating patients with interstitial lung diseases (ILDs) effectively.

The median waiting time to mortality on the waiting list was only six months, highlighting a critical factor contributing to increased waiting list mortality. While lung transplantation criteria are explicitly defined for idiopathic pulmonary fibrosis (IPF) patients, establishing uniform criteria for non-IPF ILD patients is more intricate due to the diverse etiologies within this group. As treatment options for advanced lung diseases continue to expand, patients with unique disease trajectories of ILDs are increasingly referred for lung transplantation at later stages of their illness [10]. This relatively short duration underscores the urgent need for timely access to suitable donor organs. The brevity of this waiting period suggests that patients often experience rapid deterioration in their health while on the list, which significantly impacts their survival chances. Addressing this issue requires a multi-faceted approach, including enhancing the efficiency of organ procurement and allocation processes, and improving patient management strategies to extend survival during the waiting period.

IPF remains the leading indication for lung transplantation among ILDs. The lung transplant rate for IPF ranges between 61.8% and 74.3% in other progressive lung diseases groups [1, 10]. However, a study by Bode et al. reported that 34.8% of ILD patients had IPF, aligning closely with our study’s finding of 34.5% [11]. This discrepancy suggests that IPF might be considered for lung transplantation less frequently than other ILDs or that these groups exhibit different levels of transplantation need. Factors influencing these differences may include the rate of disease progression, the patient’s response to treatment, and their overall clinical condition.

The prevalence of PH in ILDs varies depending on the disease stage. It ranges from 3.5 to 15% in the early stages, increases to 30–50% in advanced stages, and reaches 60–90% among patients listed for lung transplantation [12]. Data on the impact of PH on post-transplant prognosis in IPF are inconsistent.

Patients with PH may have had a prolonged disease course and accumulated multiple comorbidities, potentially predisposing them to poorer outcomes following transplantation. The current approach to treating Group 3 PH emphasizes managing the underlying lung disease, optimizing oxygen therapy, and addressing other contributory factors. PH-directed therapies may be considered on a case-by-case basis, particularly in patients with severe symptoms or who do not respond adequately to conventional treatments. However, this should be executed with careful consideration and in consultation with specialists who have expertise in the nuances of Group 3 PH. The findings from the INCREASE study are promising and suggest that inhaled treprostinil could be a valuable addition to the treatment for PH-ILD. The results indicate that treprostinil may help improve exercise capacity, reduce cardiac stress, and potentially slow disease progression in this challenging patient population [13]. The subgroup analysis of the STEP-IPF study suggests that sildenafil may be particularly effective in patients with IPF who exhibit right ventricular dysfunction (RVD). This targeted approach holds promise for improving the management of IPF and potentially other conditions associated with ILD, thereby enhancing patient outcomes and supporting more personalized treatment strategies [14]. Our study revealed a PH rate of 76% in patients with non-IPF ILDs. In light of recent publications, we are initiating treatment with Revatio (sildenafil) and inhaled treprostinil for patients with pulmonary hypertension who are on the waiting list. We anticipate that future studies will provide a clearer treatment algorithm for managing pulmonary hypertension in this patient population.

The utilization of intraoperative ECMO in lung transplantation is on the rise globally. Routine use of intraoperative ECMO has been associated with excellent primary graft function and favorable medium-term outcomes. These results provide strong support for the routine application of intraoperative ECMO in bilateral lung transplantation [15]. We do not routinely use intraoperative ECMO in our clinic. We have stated the indications for using ECMO in our previous study [16]. According to our experience with bronchiectasis, hemodynamic deterioration and irreversible damage to the heart may occur due to bleeding that will require massive blood transfusion during intraoperative use of ECMO in lung transplantation, especially in patient groups with chest wall adhesions. In our study, we observed significant adhesions in patient groups with silicosis, scleroderma, and sarcoidosis. One of our cases with pleuroparenchymal fibroelastosis (PPFE) presented with severe adhesions, whereas the other PPFE case showed no adhesions. During surgical exploration, we prioritize the removal of adhesions to the extent possible. After achieving hemostasis, we initiate ECMO. The use of heparin-coated ECMO lines has facilitated earlier initiation of ECMO and enhanced procedural comfort by significantly reducing our reliance on systemic anticoagulants. Studies on the potential bleeding risks associated with ECMO and strategies for managing these risks are ongoing.

In a study conducted by Perin et al., which compared patients with silicosis to those with IPF, it was observed that intraoperative complications during lung transplantation are particularly challenging in patients with silicosis due to the increased complexity of lung dissection and a greater propensity for significant bleeding. However, the study found no significant difference in length of stay between patients with IPF and those with silicosis [17]. Among the four patients with silicosis listed for lung transplantation, three underwent the procedure. The first patient who received a transplant has been followed up for 63 months without any complications. However, one patient died due to severe adhesions to both the chest wall and the hilum, which led to significant bleeding. Another patient, who had no adhesions and underwent an uneventful operation, unfortunately, died postoperatively due to hyperammonemia syndrome. The etiology of silicosis can influence the nature of the surgical procedure, as well as the formation of hilar and pleural adhesions. Postoperative periods frequently present complex challenges and complications.

**Fig. 3:**
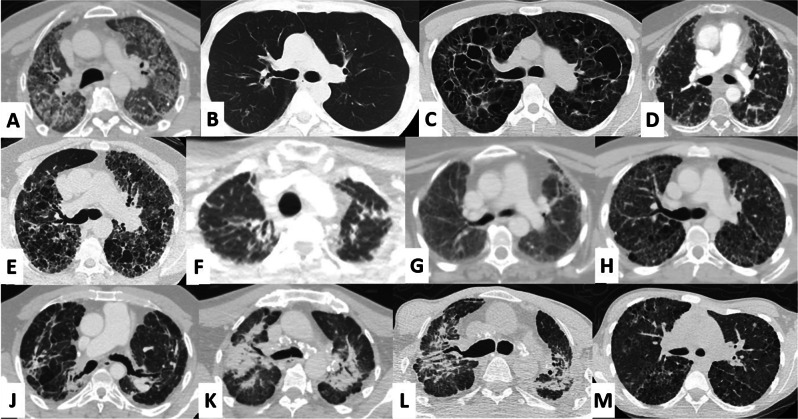
Thorax computer tomography images of some of the interstitial lung diseases other than idiopathic pulmonary fibrosis: **A** Alveolar Protinosis; **B** Graft Versus Host Disease; **C** Histiocytosis X; **D** Hypersensitivity Pneumonia; E Nonspecific Interstitial Pneumonia; **F** Pleuroparenchymal Fibroelastosis; **G** Post Covid Fibrosis; **H** Pulmonary Ossification; **J** Sarcoidosis; **F** Silicosis; **L** Scleroderma; **M** Unclassifiable

In interstitial lung disease, there may be significant discrepancies between clinical symptoms and imaging findings. For instance, patients with conditions such as Langerhans cell histiocytosis X, who may present with minimal healthy lung parenchyma on imaging, often report minimal impact on their daily activities. Conversely, some patients with minimal abnormalities visible on computerized tomography scans may be oxygen-dependent and require significant assistance with daily tasks. This highlights that a direct correlation between clinical symptoms and imaging results is not always evident. Although tomography images offer critical insights into the diagnosis and progression of the disease, we contend that evaluating the patient’s overall condition and the results of the 6 MWT may provide more precise information regarding the need for transplantation. This perspective underscores the importance of assessing clinical symptoms and their impact on daily life independently of imaging findings (Fig. 3).

Transplant listing criteria for conditions such as chronic obstructive pulmonary disease and cystic fibrosis are clearly delineated. Treatment protocols are overseen by specialists in these diseases, who adhere to these established criteria to ensure appropriate patient management and evaluation. In non-IPF ILDs, the lack of comprehensive patient profiles and the difficulty in determining disease progression can lead to significant challenges in the lung transplant referral and listing processes. The heterogeneous clinical presentations of these diseases complicate the accurate assessment of individual patient needs and prognoses, making the management and decision-making processes more complex. Rare lung diseases such as silicosis and Langerhans cell histiocytosis X are typically managed proactively to prevent progression to the stage where lung transplantation becomes necessary. As the disease advances and other treatment options are no longer effective, lung transplantation may be considered. In such cases, it is crucial to engage a specialized team and collaborate with expert health centers to optimize patient outcomes.

Non-IPF ILDs remain the predominant indication for lung transplantation within the ILD category due to their progressive nature and limited treatment options. These diseases often exhibit a relentless progression not adequately managed by current therapeutic strategies. The integration of comprehensive clinical criteria, meticulous patient selection, and advancements in transplantation techniques are continually evolving. These developments offer promising prospects for improved outcomes in individuals afflicted with these challenging conditions.

This study has several limitations that should be considered. Firstly, the relatively small number of patients included in the study limits the generalizability of the findings. Additionally, the study was retrospective and conducted at a single center, which may introduce biases and affect the applicability of the results to other settings. The heterogeneity within the patient group further complicates the interpretation of the results, as variations in patient characteristics could influence outcomes. Furthermore, the absence of a power analysis restricts our ability to determine whether the sample size was sufficient to detect meaningful differences.

## Conclusions

Lung transplantation offers substantial benefits for patients with non-IPF ILDs, potentially enhancing both quality and length of life. However, the complexities of this patient group—such as disease characteristics and comorbidities—impact eligibility, pre-transplant risks, and post-transplant care. The protocols and procedural choices of the transplant center are also crucial in determining eligibility and guiding the overall transplantation process.

## Data Availability

All data generated or analyzed during this study are included in this published article.However, if requested, we can share the requested data of all our cases with the editor and the relevant reviewers.
